# Complete genome of *Allorhizobium* (previously *Agrobacterium*) *vitis* strain CG957=AA25 from Afghanistan

**DOI:** 10.1128/mra.00468-24

**Published:** 2024-07-16

**Authors:** Han Ming Gan, Li Yuan Liew, Michael A. Savka

**Affiliations:** 1 Patriot Biotech Sdn. Bhd, Bandar Sunway, Selangor, Malaysia; 2 Program in Biotechnology and Molecular Bioscience, Thomas H. Gosnell School of Life Sciences, Rochester Institute of Technology, Rochester, New York, USA; University of Strathclyde, Glasgow, United Kingdom

**Keywords:** Afghanistan, *Agrobacterium vitis*, *Allorhizobium vitis*, crown gall disease, grapevine, nopaline *A. vitis *strain

## Abstract

Using Nanopore Q20+ sequencing, we report the complete genome of *Allorhizobium* (*Agrobacterium*) *vitis* strain CG957=AA25, isolated nearly 40 years ago from a grapevine crown gall in Afghanistan. The assembled genome size is 6 Mb, comprising a circular chromosome, a linear chromid, a Ti plasmid, and two non-Ti plasmids.

## ANNOUNCEMENT

Crown gall disease is a prevalent concern in grape-growing regions worldwide, with particular economic impact observed in areas subject to severe winter temperatures conducive to *Allorhizobium vitis* infection through wound sites (1). To investigate genetic variability patterns and the mechanisms shaping *A. vitis* speciation and adaptation, we report the genome sequencing of *A. vitis* strain CG 957 = AA25.

Strain CG957 = AA25, isolated from an Afghan nopaline-type crown gall in 1984 by Ercolani, G. L. (2), was provided by Dr. Tom Burr, Cornell University, Geneva, NY, for our research. We have preserved its glycerol stock at −80°C since then. The glycerol stock was revived on potato dextrose agar (PDA) and incubated for 24 hours at 30°C. A single colony was re-streaked onto a new PDA plate, producing pure culture for DNA extraction. Ten pure colonies were resuspended in RNA shield (Zymo Research, Irvine, CA). Following incubation at 60°C for 30 minutes, the lysate was spun down, and the supernatant was mixed with an equal volume of AmpureXP SPRI beads (Beckman Coulter, Brea, CA), followed by 5 minutes of room temperature incubation. The bead-bound DNA was washed with 70% ethanol and eluted in 50 μL of TE buffer (3).

A total of 500 ng unsheared DNA (no size selection) was processed with the SQK-LSK114 Ligation Sequencing Kit (Oxford Nanopore, UK) and sequenced on a Flongle R10.4.1 flow cell (Oxford Nanopore, UK). Base-calling and adapter trimming were performed using Dorado v0.5.1 (dna_r10.4.1_e8.2_400bps_sup@v4.3.0). The run produced 395.4 megabases from 74,332 reads (N50 length: 7,063 bp). Raw reads longer than 1,000 bp were assembled *de novo* using Flye v2.9 (4) (default settings). Circularization of the genome was performed as part of the Flye assembly and was reported without additional rotation. Maximum likelihood tree construction used the GToTree v1.4.7 pipeline (5).

**Fig 1:**
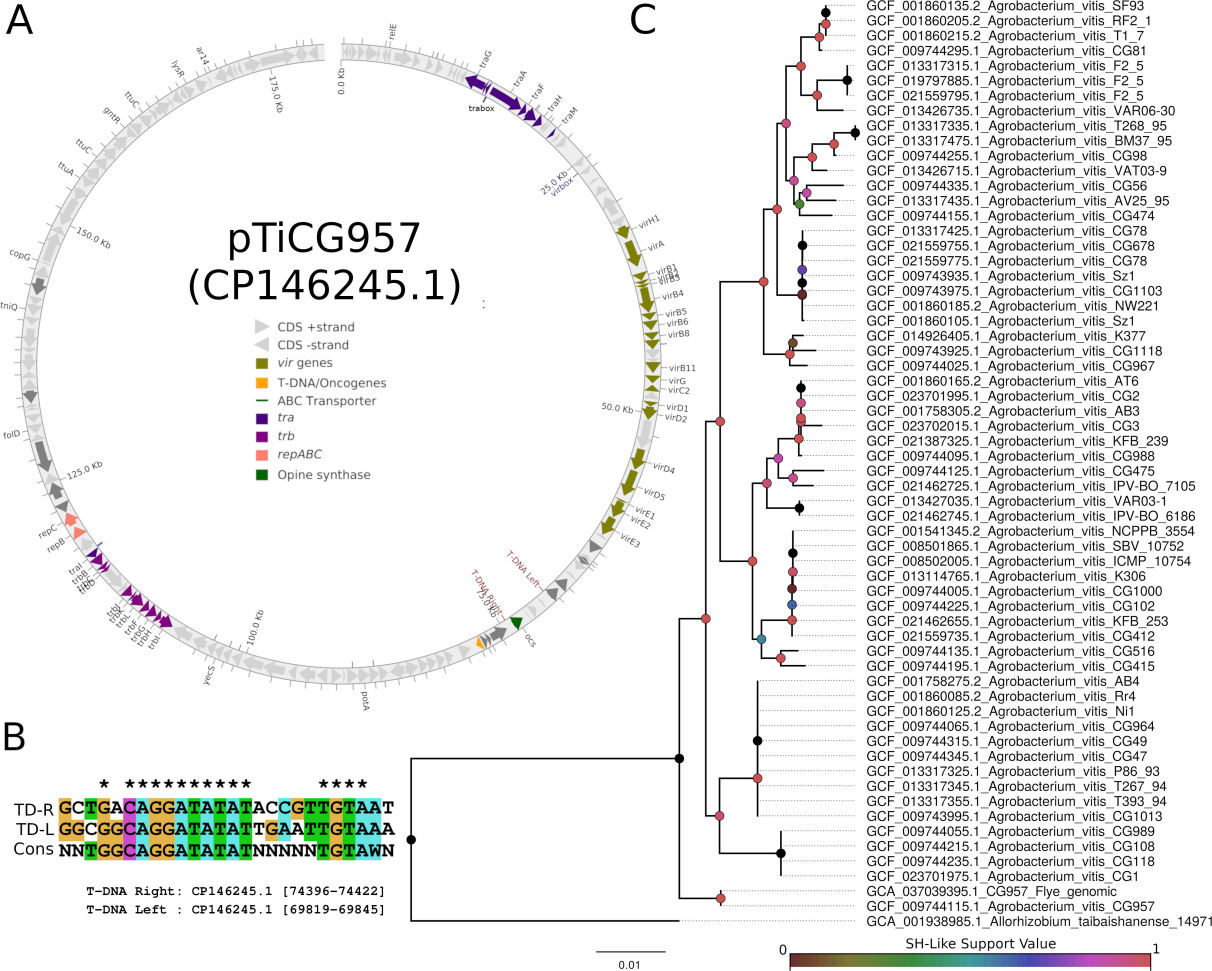
Virulence factors and evolutionary lineage of *Allorhizobium vitis* CG957 = AA25. (**A**) Putative Ti plasmid of strain CG957 = AA25, pTiCG957, with highlighted genes associated with virulence and opine synthesis as annotated by Beav v.1.1.0 (7). (**B**) Nucleotide alignment of the predicted left and right T-DNA borders. TD-R, T-DNA right border; TD-L, T-DNA left border; Cons, consensus border sequence as annotated by Beav v.1.1.0 (7). (**C**) A GTOTree-constructed maximum likelihood tree depicting the evolutionary relationships of *A. vitis* strains. Briefly, alphaproteobacterial conserved proteins were identified in the genome assemblies followed by individual alignment of each protein using MUSCLE v3.8 (8) and tree construction based on the concatenated protein alignment using FastTree v2.1.11 (9). The tree was rooted with *A. taibaishanense* as the outgroup. Branch lengths indicate the number of substitutions per site, and node colours indicate Shimodaira-Hasegawa (SH)-like support values, respectively.

The genome size of strain CG957 = AA25 is 6 Mb (58% GC content and N50 of 3.9 Mb) and was assembled into five contigs. These include a circular chromosome (chromosome I; 3,889,016 bp; 58.0% GC), a linear chromid (chromosome II; 1,229,846 bp; 58% GC), and two circular non-Ti plasmids (pUnnamed1; 536,170 bp; 57% GC, pUnnamed2; 206,864 bp; 57.5% GC), along with a circular Ti plasmid (pTiGC957; 180,253 bp; 57% GC). NCBI Prokaryotic Genome Annotation Pipeline v6.6 (6) identified 12 rRNAs, 55 tRNAs, 5,264 protein-coding genes, and 163 pseudogenes. Plasmid pTiCG957 contains the *tra* operon and *vir* operon (Fig. 1A) and two putative T-DNA arms (Fig. 1B). Two tartrate dehydrogenase genes (Protein IDs: WS83017 and WWS83022) were annotated on the Ti plasmid, providing genomic potential for tartrate utilization. Phylogenomic analysis placed strain CG957 = AA25 in a basal position within the *A. vitis* clade (Fig. 1C), reflecting its genomic divergence that is consistent with its lower than 95% average nucleotide identity (93.08% ANI, 78.44% genome coverage) to strain K309^T^ (GCF_001541345.2).

## Data Availability

Genome sequences of A. vitis CG 957 = AA25 were deposited in GenBank under the accession numbers CP146242 (chromosome I), CP146244 (chromosome II), CP146241 (plasmid pUnamed1), CP146243 (plasmid pUnamed2) and CP146245 (Ti plasmid pUnamed3). Nanopore reads were deposited and in the Sequence Read Archive under accession number SRR28041148.
